# Differential Accumulation of Anthocyanins in *Dendrobium officinale* Stems with Red and Green Peels

**DOI:** 10.3390/ijms19102857

**Published:** 2018-09-20

**Authors:** Zhenming Yu, Yinyin Liao, Jaime A. Teixeira da Silva, Ziyin Yang, Jun Duan

**Affiliations:** 1 Guangdong Provincial Key Laboratory of Applied Botany & Key Laboratory of South China Agricultural Plant Molecular Analysis and Genetic Improvement, South China Botanical Garden, Chinese Academy of Sciences, Xingke Road 723, Tianhe District, Guangzhou 510650, China; zhenming311@scbg.ac.cn (Z.Y.); honey_yyliao@scbg.ac.cn (Y.L.); zyyang@scbg.ac.cn (Z.Y.); 2 University of Chinese Academy of Sciences, No. 19A Yuquan Road, Beijing 100049, China; 3 Miki-cho post office, Ikenobe 3011-2, P.O. Box 7, Kagawa-ken 761-0799, Japan; jaimetex@yahoo.com

**Keywords:** *Dendrobium officinale*, anthocyanin, UPLC-MS-QTOF, anthocyanidin synthase

## Abstract

*Dendrobium officinale* stems, including red and green stems, are widely used as a dietary supplement to develop nutraceutical beverages and food products. However, there is no detailed information on pigment composition of red and green stems. Here, we investigated the content and composition of pigments in red and green stems by Ultra-performance liquid chromatography quadrupole time-of-flight mass spectrometry and assessed the differential accumulation of anthocyanins at the molecular level. The color of peels in red stems was caused by the presence of anthocyanins in epidermal cells unlike the peels of green stems. The glucoside derivatives delphinidin and cyanidin are responsible for the red color. Within the *D. officinale* anthocyanidin biosynthetic pathway, *DoANS* and *DoUFGT*, coding for anthocyanidin synthase and UDP-glucose flavonoid-3-*O*-glucosyltransferase, respectively, are critical regulatory genes related to the differential accumulation of anthocyanidin. These findings provide a more complete profile of pigments, especially anthocyanin, in *D. officinale* stems, and lay a foundation for producing functional foods.

## 1. Introduction

*Dendrobium officinale* (Chinese name: *Tie-pi-shi-hu*) is an edible perennial orchid plant that has commonly been employed as a functional food in China and other Asian countries for thousands of years [1]. *D. officinale* is frequently infused in juice, tea and wine as a functional liquid, but can also be chewed directly or stewed in soup, porridge and other dishes as a high-quality agricultural vegetable in dietary supplementation [2], and has been approved by the China Food and Drug Administration (http://eng.sfda.gov.cn/WS03/CL0755/). Phytochemical analyses of stems in *D. officinale* have validated the presence of antioxidants [3], which are associated with anti-angiogenesis [4], immuno-stimulating activity [5] and the attenuation of ulcerative colitis [6], biofunctional properties that are due to large amounts of polysaccharides, flavonoid, phenanthrenes, bibenzyls and polyphenolic compounds [7].

Currently, the quality of *D. officinale* is irregular throughout the course of artificial culture as a result of distinct phenotypic traits, i.e., non-clonal material [8]. Differences in the biological characters of *D. officinale* stems, such as color, size, hardness and thickness, may exhibit different health functions [9]. *D. officinale* stems are a widely used phytomedicinal organ [10]. Depending on the absence or presence of purple spots on their stems, *D. officinale* can be divided into stems with a green peel (hereafter green stems) and stems with a red peel (hereafter red stems), both of which exist widely on the current market. It is generally believed that these two phenotypes of *D. officinale* stems result from a distinct content and composition of pigments. However, information on pigment composition of the red and green *D. officinale* stems, as well as the differential regulation of anthocyanin pigmentation in *D. officinale* stems, is scanty.

The diversity of colors in higher plant organs, such as flowers, leaves, seeds and fruits, is primarily determined by the differential accumulation of anthocyanins [11], but these colors can be enhanced by co-pigmentation with colorless flavonoids. Anthocyanin is divided into three distinct lineages depending on its hydroxylation state, including pelargonidin (orange/red), cyanidin (pink/magenta) and delphinidin (purple/blue) [12]. Anthocyanin biosynthetic pathways, key enzymes as well transcription factors that affect anthocyanin content have been intensively elucidated in plants [13]. In the Orchidaceae, both chalcone synthase (CHS) and dihydroflavonol 4-reductase (DFR) have been cloned from *Bromheadia finlaysoniana* [14], CHS, DFR and a flavonoid 3′5′-hydroxylase (F3′5′H) from *Dendrobium* hybrids including ‘Sonia Earsakul’, ‘Sirin classic’, ‘Suree white’ and ‘Jasmine white’ [15] and CHS, DFR and F3′5′H from *Phalaenopsis* [16,17]. Anthocyanin synthase (ANS, EC 1.14.11.19) is responsible for converting colorless leucoanthocyanidins to colored anthocyanidins, in particular, via a conserved and well-characterized pathway in a wide range of plant species, including *Antirrhinum majus*, *Arabidopsis thaliana*, *Malus sieversii*, *Petunia hybrida*, *Punica granatum*, *Vitis vinifera* and *Zea mays* [18]. UDP-glucose flavonoid 3-*O*-glucosyl transferase (UFGT, EC 2.4.1.115), which is involved in the last dedicated step of anthocyanin biosynthesis, catalyzes the glycosylation of anthocyanidins and produces the first stable anthocyanins [19]. The transcript levels of *ANS* and *UFGT* are correlated with anthocyanin accumulation in the skin of *Vitis vinifera* grapes [20]. However, the genes coding for anthocyanin synthase (*DoANS*) and UFGT from *D. officinale*, i.e., *DoUFGT*, have not been detected yet, and no studies have attempted to quantify the expression levels among genes of the anthocyanin biosynthetic pathway in this medicinal orchid.

*D. officinale* with red and green stems are widely available and are considered in China as a traditional promising functional food [1]. Thus, the aim of the present study was to compare the pigment (flavonoids, anthocyanins, and carotenoids) profiles of red and green stems using ultra performance liquid chromatography coupled with photodiode array detection quadrupole time-of-flight mass spectrometry (UPLC-PDA-QTOF-MS) analysis. In addition, we aimed to unveil differential anthocyanin accumulation between red and green stems at the molecular level. These findings will advance our understanding of pigment content and composition in *D. officinale* stems, and will provide additional insight into the regulation of anthocyanin biosynthesis in orchids.

## 2. Results and Discussion

### 2.1. Red D. officinale Stems Exhibit High Antioxidant Activity

**Figure 1:**
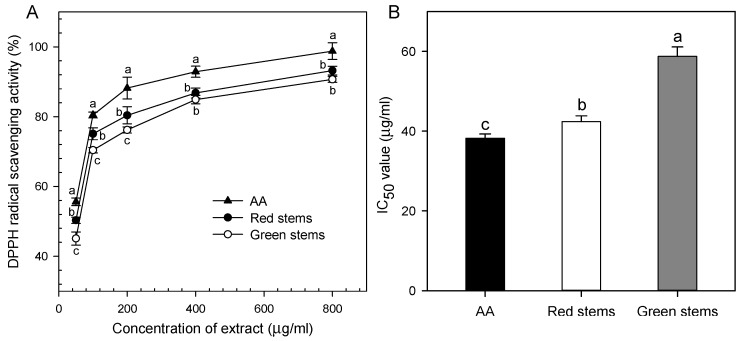
DPPH radical scavenging activity of extract from red and green *D. officinale* stems. (**A**) DPPH radical scavenging activity of AA, green and red stems extracts; (**B**) IC_50_ value of extract from red and green *D. officinale* stems. AA: ascorbic acid; DPPH: 2, 2-diphenyl-1-picryhydrazyl. Each data bar represents the mean ± standard deviation (SD; *n* = 3). Different letters above bars indicate significant differences among different treatments at *p* < 0.05 based on student’s *t*-test. Both red and green stems exhibit a significantly lower IC_50_ value according to a student’s *t*-test (*p* < 0.05) when compared with AA, and red stems demonstrate better antioxidant activities than green stems.

The antioxidant activity of *D. officinale* stems was determined by the DPPH (2, 2-diphenyl-1-picryhydrazyl) free-radical scavenging assay. As shown in Figure 1, the DPPH free-radical scavenging activity in red stems (93.5%) was slightly higher than in green stems (90.1%) when used at 800 μg/mL. Similarly, red stems showed high antioxidant activity at all concentrations. Moreover, red stems had a much lower IC_50_ value than green stems. The IC_50_ value of red stems was statistically similar to that of ascorbic acid (AA) (IC_50_ = 38.21 μg/mL) (Figure 1). Antioxidant capacity is strongly associated with anthocyanin content in pomegranate juice [21], indicating that anthocyanins contribute to antioxidant activity. These results correspond to a previous study in which *D. officinale* stems were an important source of antioxidants with 97.1% DPPH scavenging activity at 10.0 mg/mL [3], suggesting that antioxidant activity of *D. officinale* stems is likely to be related to pigment content, especially anthocyanin content.

### 2.2. Red D. officinale Stems Possess High Anthocyanin Content

**Figure 2:**
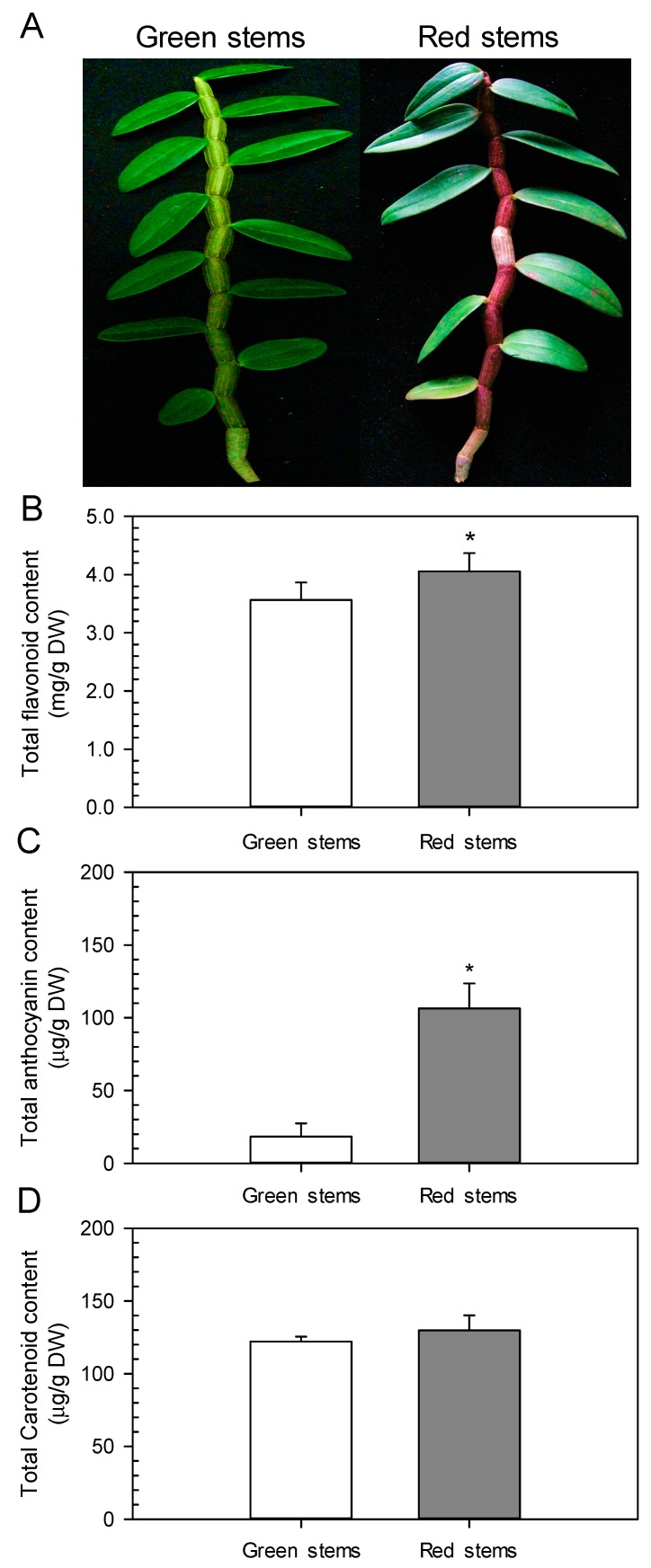
Photographs and pigment contents of *D. officinale* stems. (**A**) photographs of green stems of ‘T32-5′ and red stems of ‘T32-4′; (**B**) total flavonoid content in red and green stems; (**C**) total anthocyanin content in red and green stems; (**D**) total carotenoid content in red and green stems. Each data bar represents the mean ± SD (*n* = 3). Asterisks indicate significant differences between red and green stems according to a student’s *t*-test (*p* < 0.05). DW, dry weight.

In general, plant pigments are composed of anthocyanins, carotenoid and flavonoids [12]. Total flavonoid content in green and red *D. officinale* stems was significantly different (Figure 2). Green stems exhibited significantly lower total flavonoid content (3.6 mg/g DW) than red stems (4.1 mg/g DW). The anthocyanin content in red stems (106.4 μg/g DW) was significantly (5.85-fold, *p* < 0.01) higher than in green stems (18.2 μg/g DW). However, the carotenoid content in green stems (122.1 μg/g DW) and red stems (129.9 μg/g DW) were not significantly different. These findings indicate that anthocyanins might be responsible for the red color in red *D. officinale* stems and higher anthocyanin content corresponds to a deeper red color, which also indicates that anthocyanins are found in small amounts in green stems. A detailed composition of anthocyanins in *D. officinale* stems still has to be clearly determined. The red color in peels of *Pyrus pyrifolia* fruit is highly correlated with the content and composition of anthocyanins [22].

Other than anthocyanins, colored flavonoid glycosides contribute to the diversity of colors in fruits and flowers [13]. To date, only one study has qualitatively or quantitatively assessed the flavonoids from *D. officinale* [23]. The flavonoids in green and red *D. officinale* stems were detected by UPLC-PDA-QTOF-MS (Table S1). Multiple *O*-glycosides, including 7-*O*-[β-d-arabinopyranosyl-(1→6)-β-d-glucosyl] apigenin, lyoniresinol-3-α-*O*-β-glucopyranoside, 6-*O*-β-d-glucopyranosyl-β-D-glucopyranoside and 6-*O*-(6-deoxy-α-l-mannopyranosyl)-β-d-glucopyranoside were present in both red and green *D. officinale* stems. Furthermore, there was a strong positive correlation between DPPH free-radical scavenging activity and total flavonoid content (*R*^2^ = 0.893, *p* < 0.01). As shown in Table S1, several compounds contributing to the antioxidant pool were discovered, including paeonolide, 7-*O*-[β-d-arabinopyranosyl-(1→6)-β-d-glucosyl] apigenin and genistin 7-*O*-gentiobioside [24,25]. These results suggest that anthocyanins might play a primary function in the generation of red color of *D. officinale* stems, while the contribution of other flavonoids was secondary.

### 2.3. The Red Color of Stem Peels Is Mainly Localized in Epidermal Cells

**Figure 3:**
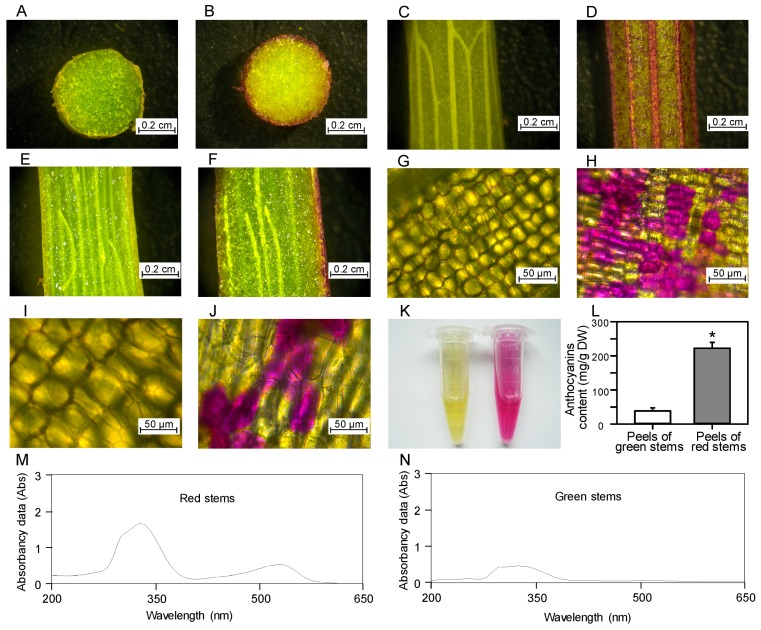
Localization and properties of pigments in *D. officinale* stems. (**A**,**C**,**E**,**G**,**I**) photograph of pigments distribution in green stems of ‘T32-5′; (**B**,**D**,**F**,**H**,**J**) photograph of pigments distribution in red stems of ‘T32-4′; (**K**,**L**) Anthocyanin extracts of peel from green and red stems; (**M**,**N**) spectrum of anthocyanin extracts from green and red stems. DW: dry weight. Asterisks indicate significant differences between peels of red stems and peels of green stems according to a student’s *t*-test (*p* < 0.05).

To further understand the function of different pigments (including anthocyanins, flavonoids, and carotenoids), the distribution of pigment-containing cells in green and red *D. officinale* stems was investigated (Figure 3). Red pigments (mainly anthocyanins) were only observed in the epidermal cells of red stems, but no red pigments were detected in green stems, corresponding to the visual phenotype of *D. officinale* stems (Figure 2). This is also related to the relatively high anthocyanin content in the peels of red stems (222.3 mg/g DW). Carotenoids might play a greater role than flavonoids in producing yellow color in *D. officinale* stems, even though carotenoid content was much lower than flavonoid content (Figure 2). The rationale is based on studies that have demonstrated that carotenoids play a major role in contributing a yellow color among a wider range of vegetables and fruits [26]. Overall, individual epidermal cells in the outer layer of green stems are arranged loosely, while epidermal cells in the outer layer of red stems are aligned tightly, and the intercellular space is small (Figure 3G,H). Furthermore, red pigmented cells showed a dot-like pattern, as seen for the epidermis of red stems (Figure 3J), but this phenomenon could not be observed in epidermal cells of green stems. After the epidermis was peeled off from green and red stems, it was found that anthocyanin content was significantly higher in the peels of red stems (222.3 mg/g DW) than in the peels of green stems (38.7 mg/g DW). The presence of pink/magenta and purple/blue in red *D. officinale* stems suggests that cyanidin- and delphinidin-based anthocyanins might accumulate [11]. The red *D. officinale* stems looked the same as red-fleshed grape berries [27], suggesting that the anthocyanin composition, including diglucosides of delphinidin, cyanidin, malvidin and peonidin, might be similar.

### 2.4. Red-Peel Color Was Determined by Delphinidin and Cyanidin Derivatives of Anthocyanins

The present study is the first to report the main composition of anthocyanins in *D. officinale* stems. As mentioned above, red and green peels of *D. officinale* stems were chiefly determined by the presence of anthocyanin-containing epidermal cells in their peels. Therefore, peels were used to analyze anthocyanin composition by UPLC-QTOF-MS. Five categories of anthocyanins, i.e., containing pelargonidin (*m*/*z* 271), cyanidin (*m*/*z* 287), peonidin (*m*/*z* 301), delphinidin (*m*/*z* 303) and malvidin (*m*/*z* 331), were identified in the peels of red and green *D. officinale* stems via precursor-ion analysis. As shown in Table 1 and Figure S1, seven anthocyanin compounds were determined. Next, we provide a summary of the differences in retention times, molecular ions, aglycone ions, main MS^2^ fragments, and the ratio of each compound in peels of red and green stems.

A scan for the precursors with *m*/*z* 303 (Figure S1A) detected delphinidin anthocyanins with molecular cations at *m*/*z* 627.1422 and 465.0942 [M-glu]^+^, which was characterized as delphinidin 3,5-*O*-diglucoside. A scan for the precursors of *m*/*z* 271 (Figure S1B,C) detected two pelargonidin anthocyanins: that with molecular cations at *m*/*z* 579.1615 was characterized as pelargonidin 3-*O*-rutinoside, while the other with molecular cations at *m*/*z* 595.1585 and 433.1092 [M-glu]^+^ was characterized as pelargonidin 3,5-*O*-diglucoside. A scan for the precursors of *m*/*z* 287 (Figure S1D,E) detected two cyanidin anthocyanins with molecular cations at *m*/*z* 449.1010 and 449.1007, which were characterized as cyanidin 3-*O*-glucoside and cyanidin 3-*O*-galactoside, respectively. A scan for the precursors of *m*/*z* 301 (Figure S1F) detected peonidin anthocyanins with molecular cations at *m*/*z* 625.1638 and 463.0942 [M-glu]^+^, which were characterized as peonidin 3,5-*O*-diglucoside. A scan for the precursors of *m*/*z* 331 (Figure S1G) detected malvidin anthocyanin with molecular cations at *m*/*z* 493.13, which was characterized as malvidin 3-*O*-glucoside. In summary, all anthocyanins were listed as presumptive structures based on their comparison with chromatographic, UV, and MS properties (Table 1). In addition, delphinidin 3,5-*O*-diglucoside, cyanidin 3-*O*-galactoside and cyanidin 3-*O*-glucoside were only detected in red stems, but not in green stems. Although the composition of anthocyanins was identified in *D. officinale* stems, these compounds were similar to banana fruit [28] and grape berries [27]. These results indicate that diglucoside or glucoside derivatives of delphinidin and cyanidin are responsible for the purple-red color of red *D. officinale* stems.

### 2.5. DoANS and DoUFGT Are Vital Regulatory Genes during Accumulation of Anthocyanidins

**Figure 4:**
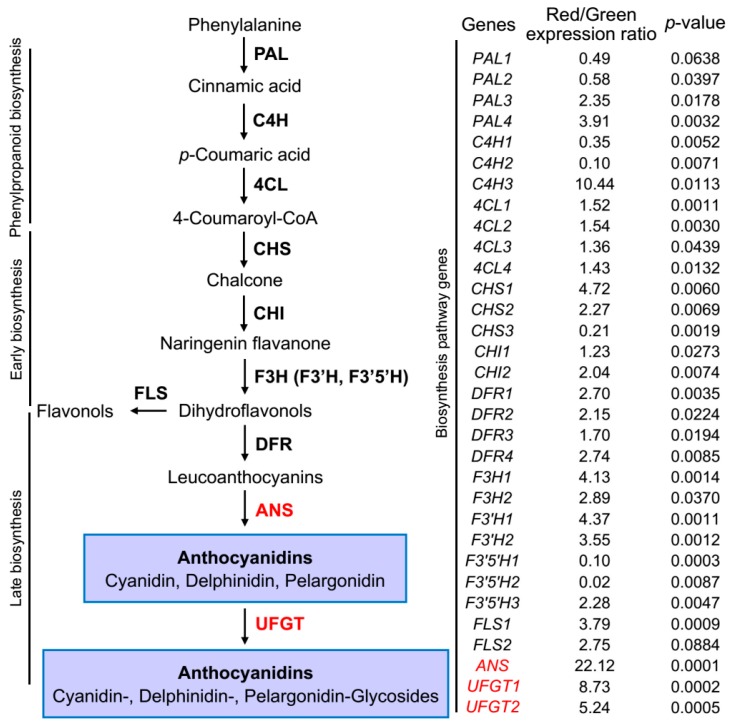
Transcript levels of genes involved in the anthocyanin biosynthesis in *D. officinale* stems. (**A**) simplified model of the anthocyanin biosynthetic pathway; (**B**) qRT-PCR analysis of the transcript abundance of the anthocyanin biosynthesis-related genes in peels of green and red stems. Red/green expression ratio is equal to the ratio between the transcript levels of red stems and the transcript levels of green stems. ANS, anthocyanidin synthase; 4CL, 4-coumarate CoA ligase; C4H, cinnamate 4-hydroxylase; CHI, chalcone isomerase; CHS, chalcone synthase; DFR, Dihydroflavonol 4-reductase; F3H, flavanone 3-hydroxylase; F3′H, flavonoid 3′-hydroxylase; F3′5′H, flavonoid 3′5′-hydroxylase; FLS, flavonol synthase; PAL, phenylalanine ammonia lyase. *p* value of expression ratio between red and green stems is calculated with a student’s *t*-test.

To further clarify the correlation of anthocyanin content with transcript levels of genes involved in anthocyanin biosynthesis, 13 anthocyanin-related genes in red and green *D. officinale* stems were detected by quantitative real-time PCR (Figure 4). The transcript levels of most genes were much higher in red stems than in green stems. Anthocyanin biosynthesis in higher plants is controlled by structural and regulatory genes, i.e., transcription factors [11]. The expression levels were in line with the distribution of anthocyanins: in red stems, the level expression of *F3H* and *F3′H* was relatively higher than in green stems, 2.89-4.13-fold and 3.55-4.37-fold, respectively. In addition, relative to green stems, *F3′5′H1* and *F3′5′H2* exhibited a significantly lower expression (0.10- and 0.02-fold, respectively) in red stems, while *F3′5′H3* was highly expressed (8.23-fold) in red stems, indicating that *F3′5′H3* might exercise a different function. The hydroxylation state of flavonoids is dominated by the enzymatic activity of F3′H and F3′5′H, corresponding to ‘red’ and ‘purple’ genes, respectively [12], caused by the generation of cyanidin (pink/magenta) and delphinidin (purple/blue)-based anthocyanins [19]. The expression levels of genes related to phenylpropanoid and early anthocyanin biosynthesis were lower in red stems, while genes related to late anthocyanin biosynthesis were higher in red stems (Figure 4). Interestingly, the abundance of anthocyanidin synthase gene (*DoANS*) transcript was 22.12-fold higher in red stems than in green stems (Figure 4). ANS, an enzyme in the anthocyanin biosynthetic pathway, catalyzes the reaction from colorless leucoanthocyanidins to colored anthocyanidins in plants [18]. The UDP-glucose flavonoid 3-*O*-glucosyltransferase (UFGT) is the final enzyme in the anthocyanin pathway, transferring a glucosyl moiety from UDP-glucose to the 3-hydroxyl group, and this process is vital for forming stable anthocyanidins [29]. In addition to *DoANS*, the same phenomenon occurred in *DoUFGT1* and *DoUFGT2*, with 8.73-fold and 5.24-fold higher levels, respectively in red stems than in green stems. Given that the high expression levels of *DoANS*, *DoUFGT1* and *DoUFGT2* in red stems are significantly positively correlated with anthocyanidin content (*R*^2^ = 0.98, 0.96 and 0.90, respectively, *p* < 0.01) (Figure 4), it is possible that they might be critical regulatory genes in anthocyanidin accumulation in red *D. officinale* stems. To date, several structural genes as well as some regulatory genes in the anthocyanidin biosynthetic pathway of the *Dendrobium* genus have been isolated [7,30,31], but no reports exist on *DoANS*, *DoUFGT1* and *DoUFGT2* in this pathway of *D. officinale*.

A target sequence (Unigene0101519) coding for anthocyanidin synthase (EC: 1.14.11.19) was mined from the *D. officinale* transcriptome [32]. Nested PCR yielded the full-length cDNA of *DoANS*, and possessed a 1077-bp open reading frame (ORF) and a polypeptide of 358 amino acids, which was submitted to NCBI (GenBank accession number MH458949). Just as important, *DoUFGT1* and *DoUFGT2* were isolated and submitted to NCBI, with a 1359-bp ORF coding for 452 amino acids, and a 999-bp ORF coding for 332 amino acids, respectively, which were also submitted to NCBI (GenBank accession number MH663506 and MH663505, respectively). Phylogenetic analysis revealed that DoANS was evolutionarily closer to monocotyledonous plants than to dicotyledonous plants (Figure S2). Multi-alignment by ClustalX suggested that DoANS was phylogenetically closer to *Oncidium hybridum* (77.99% identity) and *Cymbidium dayanum* (84.12% identity). Moreover, a highly conserved 2OG-Fe(II) oxygenase domain, which is a typical ANS binding site [33], as well as three critical binding sites (ferrous-iron coordination, substrate binding site and a binding site of 2-oxoglutarate) were strictly conserved in DoANS (Figures S3 and S4), as well as in most well-characterized ANS proteins in plants such as *Arabidopsis thaliana* [33], *Oncidium* Gower Ramsey [30], *Pyrus pyrifolia* [22], *Solanum melongena* [34] and *Vitis vinifera* [20]. As for DoANS, a plant secondary product glycosyltransferase (PSPG) box (belonging to the UDP-glucuronosyltransferase family) was conserved in DoUFGT1 and DoUFGT2 (Figure S5). Unstable anthocyanidins are stabilized by glycosylation at the 3-*O*-position or the 5-*O*-position by UF3GT and UF5GT, respectively, and then transported to acidic vacuoles, which is essential for coloration [13]. Phylogenetic analysis revealed that DoUFGT1 was clustered in the UF3GT group, while DoUFGT2 was clustered in the UF5GT group (Figure S6). As shown in Figure 4, *DoUFGT1* exhibited higher transcript levels in red stems than *DoUFGT2*, which corresponded well with the higher number of anthocyanidin compounds with a 3-*O*-position (Table 1). Thus, DoUFGT was thought to be the key enzyme for anthocyanin biosynthesis in *D. officinale*. *SmFAS*, which encodes anthocyanidin synthase, is a putative candidate gene responsible for the purple-color peels of *Solanum melongena* L. [34]. In addition, UFGT might strongly affect the coloration of grapevine peels [20]. Taken together, the expression of *DoANS* and *DoUFGT* was determined to be much higher in red stems than in green stems, which coincides with the results observed in peel color and anthocyanin content, indicating the role of *DoANS* and *DoUFGT* in regulating the accumulation of anthocyanins.

## 3. Materials and Methods

### 3.1. Chemicals and Regents

All reagents or solvents used were of analytical, HPLC or HPLC-MS grade. Absolute ethanol, acetone, aluminum nitrate nonahydrate (Al(NO_3_)_3_·9H_2_O), hydrochloric acid (HCl), L-ascorbic acid, sodium hydroxide (NaOH), and sodium nitrite (NaNO_2_) were obtained from Sinopharm Chemical Reagent Co., Ltd. (Shanghai, China). The 2,2-diphenyl-1-picrylhydrazyl (DPPH) radical, formic acid, leucine enkephalin and methanol, as well as two standards, rutin and cyanidin 3-*O*-glucoside, were purchased from Sigma-Aldrich (St. Louis, MO, USA). The iTaq^TM^ Universal SYBR^®^ Green Supermix was purchased from Bio-Rad Laboratories (Hercules, CA, USA).

### 3.2. Plant Materials

Green stem *D. officinale* ‘T32-5′ (mainly distributed in sheltered and moist places in forests) and red stem *D. officinale* ‘T32-4′ (epiphytes growing on rocks in sunlight) were sampled under natural conditions in a greenhouse of the South China Botanical Garden, Chinese Academy of Sciences, Guangzhou, China (23°10′ N, 113°21′ E). Ten stems were gathered from an individual potted plant, and 10 independent plotted plants were sampled for each stem type. The plants were two years old. A total of 200 stems (including 100 green stems and 100 red stems, with a uniform size approximately 20 cm in height and 0.5 cm in diameter) were collected in a ready-to-eat stage (12 months after seedlings were transplanted), which is the optimum harvest time according to the Chinese pharmacopoeia [10]. Half of the green and red stems were stored separately at −80 °C in a refrigerator (Haier Group Co., Qingdao, China) until the analyses of metabolites. To determine the content of total flavonoids, anthocyanins and carotenoids, the remaining stems were oven dried, ground into powder with a diameter less than 0.3 mm, and stored in a vacuum pack (VIP320, Beijing Torch SMT Inc. Co., Beijing, China) at 4 °C.

### 3.3. Assessment of Total Flavonoids, Anthocyanins and Carotenoids Content in D. officinale Stems

Total flavonoid content was detected by a colorimetric method [28] under natural light at room temperature with minor modifications. Briefly, powdered oven-dried stems (1.0 g) were extracted in an sxt-06 Soxhlet extractor (Hangzhou Chincan Trading Co., Hangzhou, China) with 20 mL of 80% aqueous ethanol for 1 h at 80 °C, and the residues were re-extracted by the same solvent. Both supernatants were pooled and insoluble material was removed by a 0.45 μm filter (jtsf0311, Tianjin Jinteng Experiment Equipment Co., Ltd., Tianjin, China) until a final volume of 50 mL was obtained. Extract (0.5 mL) was transferred to a 10-mL plastic tube containing 0.3 mL NaNO_2_ (5%, *w*/*v*) for 5 min. Then, 0.3 mL Al(NO_3_)_3_·9H_2_O (10%, *w*/*v*) was added. After incubation for 6 min, the reaction was terminated by adding 2 mL of 1 m NaOH, and further diluted with 30% aqueous ethanol up to 10 mL. A standard curve was calibrated using diluted rutin with 30% aqueous ethanol (0–400 μg/mL). Absorbance at 510 nm was recorded with a UV-6000 spectrophotometer (Shanghai Metash Instruments Co., Shanghai, China). Total flavonoid content was quantified as mg of rutin equivalent per gram dry weight (DW).

Total anthocyanin content of green and red stems was measured by a pH differential protocol [35]. Detailed information is provided in the Supplementary Information.

Total carotenoid content was determined using the protocol of Lichtenthaler and Wellburn [36]. Detailed information is described in the Supplementary Information.

### 3.4. Compositional Analysis of Flavonoids and Anthocyanins by UPLC-QTOF-MS

Compositional analysis of flavonoids and anthocyanins in red and green stems of *D. officinale* was identical to a previously reported procedure [28]. The detailed protocol is described in the Supplementary Materials and methods (Supplementary Information).

### 3.5. Evaluation of Antioxidant Activity In Vitro

Antioxidant activity in vitro was determined using a DPPH free-radical scavenging test following Luo et al. [37]. Briefly, 0.1 mL of extract at five concentrations (50, 100, 200, 400 and 800 μg/mL) was separately mixed with 3.9 mL of DPPH solution (0.1 mmol/L in methanol). The reaction mixture was shaken vigorously and placed in the dark at 25 °C for 30 min. The decolorization of DPPH was monitored at 517 nm with a UV-6000 spectrophotometer (Shanghai Metash Instruments Co., Shanghai, China). DPPH radical scavenging activity (%) was determined using the following equation: DPPH radical scavenging activity (%) = 1 − (A_sample_ − A_control_)/A_blank_ × 100, where A_blank_ is the absorbance of the control (DPPH solution without extract), A_control_ is the absorbance of the extract without DPPH, and A_sample_ is the absorbance of the extract with DPPH. The activity of the extract was expressed as 50% inhibition concentration (IC50, μg/mL), or half of radical scavenging activity, which was calculated using a forecast linear regression in Excel 2013 (Microsoft Co., Redmond, WA, USA). Ascorbic acid solution (identical concentration as extract) was used as the positive control.

### 3.6. Light Microscopy of D. officinale Stem

To determine pigment distribution in *D. officinale* stems, green and red stems were cut by hand using a razor blade (AS-D2, Shanghai Razor Blade Co., Ltd., Shanghai, China). Samples were mounted on glass slides (BM5116, SailBoat Lab Co., Ltd., Ningbo, China) and covered with a drop of distilled water. Epidermal micro-morphology was visualized using a Leica DM1000 LED microscope (Leica, Wetzlar, Germany) with bright-field illumination.

### 3.7. Gene Expression Analysis by Quantitative Real-Time PCR

RNA was extracted and cDNA was prepared from *D. officinale* stems according to Yu et al. [38]. The gene-specific primers for anthocyanin biosynthesis listed in Table S2 were designed by Primer Premier 5.0 (Premier Biosoft International, Palo Alto, CA, USA). Transcript abundance of the anthocyanin biosynthesis-related genes were analyzed on an ABI 7500 real-time PCR instrument (Applied Biosystems, Foster City, CA, USA), using iTaq^TM^ Universal SYBR^®^ Green Supermix (Takara Bio Inc., Kyoto, Japan) in accordance with the manufacturer’s protocol. *D. officinale* actin (GenBank accession: JX294908) was used as an internal standard to quantify gene targets. Relative RNA levels were calculated using the 2^−ΔΔ*C*T^ method [39].

### 3.8. Cloning of Genes and Phylogenetic Analysis

Based on our reported *D. officinale* transcriptome [32], a target sequence (Unigene0101519) coding anthocyanidin synthase, designated as *DoANS*, was amplified from cDNA of red and green *D. officinale* stems separately by using the Premix HS PrimeSTAR HS DNA Polymerase kit (Takara Bio Inc., Dalian, China) with specific primers (Table S2). Likewise, *DoUFGT1* (Unigene0117481) and *DoUFGT2* (Unigene0142333) were individually cloned from red and green stems. Thermal cycling was carried out as follows: 94 °C for 3 min, 30 cycles of 94 °C for 30 s, 55 °C for 30 s, and 72 °C for 1 min, and then 72 °C for 10 min. Purified PCR products were sub-cloned into pMD18-T vector (Takara Bio Inc., Dalian, China) and then sequenced at the Beijing Genomics Institute (BGI, Shenzhen, China).

The amino acid alignment of DoANS and its homologues including those of *Allium cepa*, *Arabidopsis thaliana*, *Brassica juncea*, *Brassica napus*, *Cymbidium dayanum*, *Lycoris chinensis*, *Oncidium hybridum*, *Prunus salicina*, and *Vitis vinifera* obtained from the National Center for Biotechnology Information (NCBI, http://www.ncbi.nlm.nih.gov) were performed using ClustalW [40]. In addition, a rooted phylogenetic tree was generated with MEGA software (5.05, Lynnon Biosoft, Foster City, CA, USA) [41] by the neighbor-joining (NJ) method and using the Jones–Taylor–Thornton (JTT) model with 1000 bootstrap replicates.

### 3.9. Statistical Analysis

All analyses were conducted in triplicate. Data were analyzed using SPSS version 22.0 software (IBM Corp., Armonk, NY, USA). Statistical analysis of metabolites in green and red stems was performed by one-way analysis of variance (ANOVA) with mean separation by a student’s *t*-test (*p* < 0.05). The level of statistical significance was set at *p* < 0.05. Correlation analysis was performed with Pearson’s correlation coefficient (*R*^2^) at *p* < 0.01. Graphs were generated with SigmaPlot 12.5 (Systat Software Inc., San Jose, CA, USA).

## 4. Conclusions

**Figure 5:**
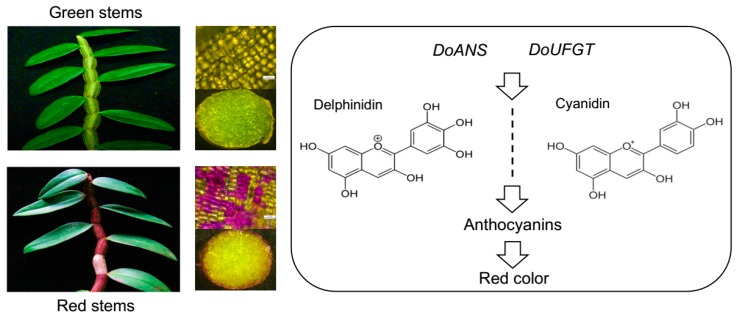
A summary of the differential anthocyanin accumulation in red and green *D. officinale* stems. *D. officinale* stems have green or red peels, and red stems are caused by the accumulation of anthocyanins. The red color is dominated by anthocyanin derivatives, delphinidin and cyanidin, which mainly exist in anthocyanin-containing epidermal cells. In addition, *DoANS* and *DoUFGT* are vital regulatory genes in anthocyanidin accumulation; *DoANS*, the gene coding anthocyanidin synthase from *D. officinale*; *DoUFGT*, the gene encoding UDP-glucose flavonoid 3-*O*-glucosyltransferase from *Dendrobium officinale*.

As shown in Figure 5, pigment content, composition, and distribution in green and red *D. officinale* stems have been investigated. A significantly higher content of anthocyanins (5.82-fold) was observed in red stems relative to green stems. The color of red stems is primarily determined by anthocyanin-containing epidermal cells in the stem peel. Among anthocyanins that were detected, delphinidin 3,5-*O*-diglucoside and cyanidin 3-*O*-glucoside are likely responsible for the red peel color. These two phenotypes based on anthocyanidin accumulation in green and red *D. officinale* stems may be controlled by *DoANS* and *DoUFGT*, which are well correlated with the amount of anthocyanidins, and reflect a highly homologous level with reported proteins involved in anthocyanidin biosynthesis. In addition, apigenin and genistin 7-*O*-gentiobioside are cardinal components in flavonoids, with a consistently high content of total flavonoids in red *D. officinale* stems, which provides them with high antioxidant activity. This information contributes to a broader understanding of the formation and pigmentation of *D. officinale* stems, and is thus closer to the ability to create overproduced anthocyanin-based functional foods.

## Figures and Tables

**Table 1:** UPLC-QTOF-MS identification of anthocyanins in *D. officinale* stems.

No.	Identification and Tentative Identification	Molecule	Rt (min)	ESI (+) MS/MS^2^ (*m*/*z*)	Red/Green Peak Area Ratio
1	Delphinidin 3,5-*O*-diglucoside	C_27_H_31_O_17_	3.62	627.1422([M]^+^)/465.0927([M-glu]^+^); 303.0477([M-2glu]^+^)	+
2	Peonidin 3,5-*O*-diglucoside	C_28_H_33_O_16_	4.54	625.1638([M]^+^)/463.0942([M-glu]^+^); 301.0627([M-2glu]^+^)	18.66 (*p* = 7.64 × 10^−9^)
3	Cyanidin 3-*O*-galactoside	C_21_H_21_O_11_	4.93	449.1007([M]^+^)/287.0515([M-gala]^+^)	+
4	Cyanidin 3-*O*-glucoside	C_21_H_21_O_11_	5.07	449.1010([M]^+^)/287.0521([M-glu]^+^)	+
5	Pelargonidin 3,5-*O*-diglucoside	C_27_H_31_O_15_	5.26	595.1585([M]^+^)/433.1092([M-glu]^+^); 271.0602([M-glu]^+^)	−
6	Pelargonidin 3-*O*-rutinoside	C_27_H_31_O_14_	5.46	579.1615([M]^+^)/271.0587([M-rutin]^+^)	33.85 (*p* = 1.35 × 10^−6^)
7	Malvidin 3-*O*-glucoside	C_23_H_25_O_12_	7.11	493.13([M]^+^)/331.0772([M-glu]^+^)	8.53 (*p* = 2.28 × 10^−5^)

Red/green peak area ratio is equal to the ratio of (the peak area of red stems) to (the peak area of green stems). + indicates that the compound was only detected in red stems, but not in green stems. − indicates that the compound was only detected in green stems, but not in red stems. ESI: electron spray ionization; MS: mass spectrometry; Rt: retention time; UPLC-QTOF-MS: ultra-performance liquid chromatography/quadrupole time-of-flight mass spectrometry.

## Supplementary Materials

Supplementary materials can be found at http://www.mdpi.com/1422-0067/19/10/2857/s1.

## Abbreviations

AA	ascorbic acid
ANS	anthocyanin synthase
4CL	4-coumarate CoA ligase
C4H	cinnamate 4-hydroxylase
CHI	chalcone isomerase
CHS	chalcone synthase
DFR	dihydroflavonol 4-reductase
DoANS	gene encoding anthocyanin synthase from *Dendrobium officinale*
DoUFGT	gene encoding UDP-glucose flavonoid 3-*O*-glucosyltransferase from *Dendrobium officinale*
DPPH	2,2-diphenyl-1-picryhydrazyl
DW	dry weight
F3H	flavanone 3-hydroxylase
F3′H	flavonoid 3′-hydroxylase
F3′5′H	flavonoid 3′5′-hydroxylase
FLS	flavonol synthase
GC-MS	gas chromatography-mass spectrometry
ORF	open reading frame
PAL	phenylalanine ammonia lyase
qRT-PCR	quantitative real time polymerase chain reaction
SD	standard deviation
UFGT	UDP-glucose flavonoid 3-*O*-glucosyltransferase
UPLC-QTOF-MS	Ultra-performance liquid chromatography/quadrupole time-of-flight mass spectrometry
