# Supplementary material for: Differential Accumulation of Anthocyanins in Dendrobium officinale Stems with Red and Green Peels
Source: Int J Mol Sci. 2018 Sep 20;19(10):2857. doi: 10.3390/ijms19102857 (PMC6212978; doi:10.3390/ijms19102857)
Supplement: Supplementary file 1 [file ijms-19-02857-s001.pdf]

SUPPLEMENTARY INFORMATION

Differential accumulation of anthocyanins in *Dendrobium officinale* stems with red and green peels

Zhenming Yu [1,†], Yinyin Liao [1,2,†], Jaime A. Teixeira da Silva [3], Ziyin Yang [1,2], Jun Duan [1,2,*]

[1] Guangdong Provincial Key Laboratory of Applied Botany & Key Laboratory of South China Agricultural Plant Molecular Analysis and Genetic Improvement, South China Botanical Garden, Chinese Academy of Sciences, Xingke Road 723, Tianhe District, Guangzhou 510650, China; zhenming311@scbg.ac.cn (Z.Y.); honey_yyliao@scbg.ac.cn (Y.L.); zyyang@scib.ac.cn (Z.Y.)

[2] University of Chinese Academy of Sciences, No. 19A Yuquan Road, Beijing 100049, China

[3] P. O. Box 7, Miki-cho post office, Ikenobe 3011-2, Kagawa-ken, 761-0799, Japan; jaimetex@yahoo.com (J.T.)

† These authors contributed equally to this work.

* Correspondence: duanj@scib.ac.cn; Tel./Fax: +86-20-38072978

Materials and methods

1. Assessment of total flavonoids, anthocyanins and carotenoids in D. officinale stems

Total carotenoid content was determined according to previous reference [1,2]. Carotenoids were released from *D. officinale* stems (2.0 g) by cold 80% acetone, homogenized in the dark, and centrifuged at 10000×*g* for 10 min. The resulting supernatant was spectrophotometrically assayed at 470, 646, and 663 nm with an UV-6000 spectrophotometer (Shanghai Metash Instruments Co., Shanghai, China). Total carotenoid content (μg/g) was quantified according to the following equations:

Chlorophyll $a = 12.21 \times A_{663nm} - 2.81 \times A_{646nm}$;

Chlorophyll $b = 20.13 \times A_{646nm} - 5.03 \times A_{663nm}$;

Carotenoids = $(1000 \times A_{470nm} - 3.27 \times \text{chlorophyll } a - 104 \times \text{chlorophyll } b)/229$.

2. Compositional analysis of flavonoid and anthocyanin with UPLC-QTOF-MS

Compositional analysis used the reported protocol [3]. *D. officinale* green and red stems were pulverized into a fine powder with liquid nitrogen using a mortar and pestle. Powdered sample (1.0 g) was accurately weighed, exhaustively extracted by vortexing with 4 mL of 70 % aqueous methanol (containing 0.1 % formic acid) for 1 min, and followed by a 40 Hz ultrasonic extraction (SB-5200D, Ningbo Scientz Biotechnology Co., Ningbo, China) in ice-cold water for 30 min. The mixture was centrifuged at 10000×*g* for 10 min and the supernatant was subjected to UPLC-QTOF-MS analysis. "Extracts were analyzed at 40 °C using the Waters ACQUITY UPLC System (Waters Corp., Milford, MA, USA) equipped with an ACQUITY UPLC HSS T3 column (100 *Å*, 2.1×100 mm, 1.8 μm, Waters, Corp., USA)." Flavonoids were detected at 350 nm while anthocyanins were determined at 520 nm. Each flavonoid or anthocyanin was quantified based on absolute peak area. The mobile phase consisted of aqueous formic acid (0.1 % in ultra-pure water, solvent A) and formic acid in acetonitrile (0.1 %, v/v, solvent B). The initial condition was 90 % solvent A and 10 % solvent B. "The following linear gradient elution was performed: 0-12 min, 10-14 % B; 12-17 min, 14-20 % B; 17-25 min, 20-30 % B; 25-25.1 min, 30-90 % B; 25.1-30.1 min, 90 % B." The flow rate and injection volume were set at 0.3 ml/min and 5 μl, respectively. Mass spectrometry was performed with a Xevo G2-XS QTOF (Waters MS Technologies, Manchester, UK) equipped with an electrospray ionization source and controlled by MassLynx software version 4.1 (Waters Corp., Milford, MA, USA). "Full MS scan was carried out in the range of *m*/*z* 100-1500 Da at a scan time of 0.5 s." Cone voltage and capillary voltage were set at 40 V and 2500 V, respectively. De-solvation temperature and source temperature were 350 °C and 100 °C, respectively. Nebulizer and de-solvation gas were nitrogen. The flow rates of cone and de-solvation gas were 50 L/h and 650 L/h, respectively. "The low energy was set as 6 V, and high energy increased from 20 V to 35 V." Leucine enkephalin (2 ng/μl, Sigma-Aldrich, St. Louis, MO, USA) was used as the lock mass, with *m*/*z* 554.2615 for the negative mode and *m*/*z* 556.2766 for the positive mode.

Table S1 UPLC-QTOF-MS identification of flavonoid compounds identified from pooled *D. officinale* green and red stems.

Adducts	*m/z*	Retention time (min)	Max fold change	Accepted Description	Formula
M+H	281.08	0.61	2.52	5-Hydroxy-2-[(2R,3S)-3-phenyl-2-oxiranyl]-4H-chromen-4-one	$C_{17}H_{12}O_4$
M+Na	421.11	5.46	56.84	4-*O*-sinapoylquinic acid	$C_{18}H_{22}O_{10}$
M+Na	483.15	5.46	84.64	Paeonolide	$C_{20}H_{28}O_{12}$
M+H, M+Na	381.24	14.32	2.79	16-Butyl-3-methoxy-estra-1,3,5(10)-triene-16-β,17-β-diol	$C_{23}H_{34}O_3$
M+H, M+Na	395.26	17.25	3.69	Cervonoyl ethanolamide	$C_{24}H_{36}O_3$
M+H, M+Na	363.26	20.42	3.01	12-[(Cyclohexylcarbamoyl)amino] dodecanoic acid	$C_{19}H_{36}N_2O_3$
M-H, M+Cl	359.11	2.63	3.54	Acalyphin	$C_{14}H_{20}N_2O_9$
M-H	623.16	4.55	12.41	Chrysoeriol 7-*O*-gentiobioside	$C_{28}H_{32}O_{16}$
M-H	563.14	4.71	3.65	7-*O*-[β-D-arabinopyranosyl-(1→6)-β-D-glucosyl] apigenin	$C_{26}H_{28}O_{14}$
M-H	595.16	4.73	11.89	Butrin	$C_{27}H_{32}O_{15}$
M-H	593.15	4.95	3.35	Genistin 7-*O*-gentiobioside 6-*O*-β-D-glucopyranosyl-β-D-glucopyranoside	$C_{27}H_{30}O_{15}$
M-H	563.14	5.18	3.99	7-*O*-[β-D-arabinopyranosyl-(1→6)-β-D-glucosyl]apigenin	$C_{26}H_{28}O_{14}$
M-H	577.16	5.46	13.34	1,3,6-trihydroxy-2-methyl-9,10-anthraquinone-3-*O*-α-L-rhamnopyranosyl -(1→2)-β-D-glucopyranoside	$C_{27}H_{30}O_{14}$
M-H	565.19	5.59	2.51	3-(β-D-Glucopyranosyloxy)-5-[(Z)-2-(4-methoxyphenyl)vinyl]phenyl β-D-glucopyranoside	$C_{27}H_{34}O_{13}$
M-H	607.16	5.81	348.34	5,7-Dihydroxy-2-(4-methoxyphenyl)-4-oxo-4H-chromen-3-yl 6-*O*-(6-deoxy-α-L-mannopyranosyl)-β-D-glucopyranoside	$C_{28}H_{32}O_{15}$
M-H, M+Cl	581.22	5.98	39.11	(-)-Lyoniresinol-3-α-*O*-β-glucopyranoside	$C_{28}H_{38}O_{13}$

Table S2 Primers used for quantitative real-time PCR (qRT-PCR) analysis in this study.

Gene	Gene ID	Forward (5'→3')	Reverse (5'→3')
PAL1	Unigene0108630	CTACATGAAGGTGGCGAAGAA	CTTGGTTGCTGCACGAATTAC
PAL2	Unigene0016044	GGTTTCAAGGGAGCAGAGATAG	CGCTCTGCACATGGTTAGT
PAL3	Unigene0158609	TACCTTGTCGGAATGCTCAC	CTCCCATCCGAGATGAGATAGA
PAL4	Unigene0103006	TTCGCTGCAGTCAGAACTC	GACTAGGCATTATTCGGGTCAA
C4H1	Unigene0158049	CAACTCCTCCCTCCAATCATTTA	GGGCAGTTCAGTCTCCATATAC
C4H2	Unigene0083313	CCAGTTTCTTCCTCTCCTCAAC	TACGGCGATTTCATCCCTATTC
C4H3	Unigene0125244	GAAGTTCGGCGATATCCTTCTC	CAAATTCGACTCCCTGGGTATG

4CL1	Unigene0112398	TAGAGAGGGACTTGATGGAGAG	CCCTCCGAAATCCTCAAACA
4CL2	Unigene0118305	CCAGGATGGGTCGATAGAATTT	GCTGGTCTACGTGGTTGATAG
4CL3	Unigene0015559	CATCATCACCCACTCCATCTT	TGAACGGAAGGCAGGATTT
4CL4	Unigene0131993	AGTGAGGACTACTCCCTTAACC	ATTCGCCTCTCCCTCCATTA
CHS1	Unigene0150238	GCTCGTGGTGCAGAATATGA	AGGCAAGAGGTCGCAATAAG
CHS2	Unigene0076676	GTAGGCGAGTGAGTTGGTAATC	CCAGACGAGCCATCAAAGAA
CHS3	Unigene0134375	GCTGGTGGTGCAGAAGATTA	CAAGACATAGTGGTCACCGAAG
CHI1	Unigene0116452	GGCTCACAATATGGCGTACA	GTGCTTCTTCTTCCTCCTCTTC
CHI2	Unigene0116451	GTGCTTCTTCTTCCTCCTCTTC	GGCTCACAATATGGCGTACA
DFR1	Unigene0104804	CAGCCCTCAGCTCTATTTCTAC	CGAGGATGTTCCGGCTTATT
DFR2	Unigene0157250	GAGTGAAGACCATCCCAAGTT	CGTCCATAGGTTTGAGGAAGAG
DFR3	Unigene0149794	CGAGAGAGATAGAGAGAGAGAGATG	TGTTCCGTTGACCTTGTATCC
DFR4	Unigene0022383	GAGATAGCAGCAAGAGAGAATGG	TCACAAGCCAGGAAGCAATAA
F3H1	Unigene0124945	GAGTACAGCGCCAAGCTAAT	CGACCAATTTCTGGTCCATTTC
F3H2	Unigene0109511	TCAACGACCACAACTCATTCA	CTTCTCTCCTCTTCCGACAAATC
F3'H1	Unigene0142709	TACTCGCAGAATGGGCTTTAG	CTTGGAGCAGAGGAAGGTTAG
F3'H2	Unigene0127406	CCGGAAGGCTAATGTCTCATC	GACGGCTACCTCATTCCTAAAG
F3'5'H1	Unigene0103961	GGGCTTCATCTCTGACCTATTC	GTCGGAGGATTGATGGGTTT
F3'5'H2	Unigene0138824	CTGAATCCGCACAAGAGTTAAAG	GGAGCCAAGAAAGGGAAGAA
F3'5'H3	Unigene0147368	CCGGCGATTAGATCCTGTATTAT	GTTGACGTCCTCCTTCAGTT
FLS1	Unigene0157397	AATCAACCCAGGCCTTCTTC	TCGCAGGAGGAGAAGGATAA
FLS2	Unigene0109511	AGCTTAGGGCTCGATGAAAG	GGAGTATCCGAATGAGCAGATAG
ANS	Unigene0101519	CTCCTTACTCTCTCTCGGTCTC	CAGCGTGGGTAGTAGTTGATTT
UFGT1	Unigene0142333	TCTTCCGGCCTCGTACTATT	GGTGACCTGGCCGTTATTT
UFGT2	Unigene0117481	GGGTTCCCGTTAGCGTTATT	GGAGTTTGTCAGCATGAGTAGG
MYB1	Unigene0012491	GGCTTCTCGGATGAAGAAGATAG	CAGGAAGATGCGCAGCTATAA
MYB2	Unigene0101304	AGGCTCAGGTGGACAAATTAC	TGTTACCAAGCAGAGCATGAA
MYB3	Unigene0116668	CTGTTCCTCCTGCATGCTAAA	CCTAACAAAGCTGGCCTACAA
MYB4	Unigene0135347	CTGCAGGCTTAGGTGGATAAA	CTGAGACCATCTGTTTCCAAGA
MYB5	Unigene0155688	AGAACTCCATGCTGTGATAAGG	CCACCTGTTCCATACTTCTCTATG
Actin	JX294908	TCCCAAGGCAAACAGAGAAA	GGCCACTAGCATATAGGGAAAG

D. officinale actin (GenBank accession no. JX294908) was obtained from NCBI. Primer Premier 5.0 software (Premier Biosoft International, Palo Alto, CA, USA) was used to design primers for this study.

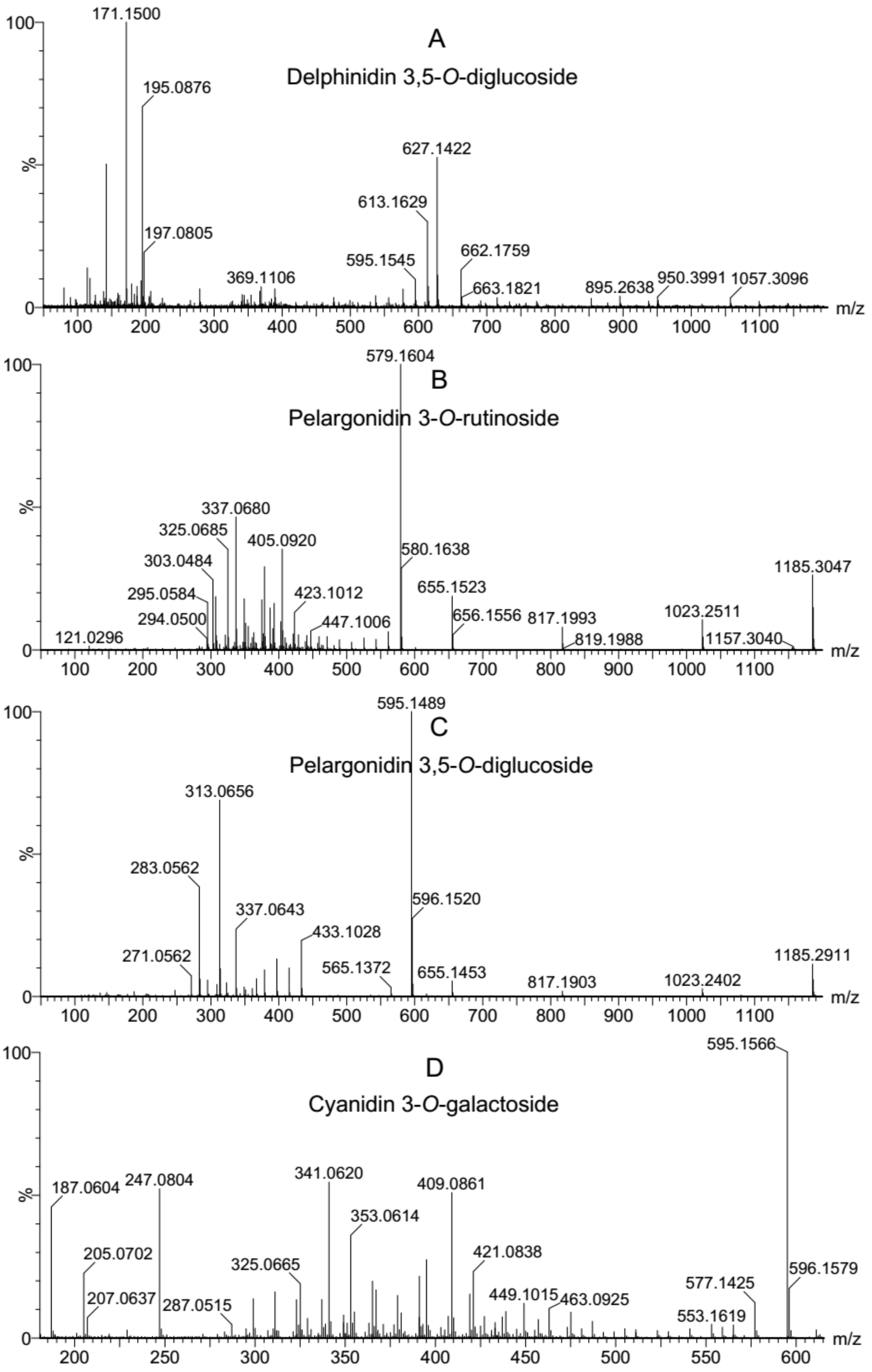

A
Delphinidin 3,5-O-diglucoside
171.1500
195.0876
197.0805
369.1106
595.1545
613.1629
627.1422
662.1759
663.1821
895.2638
950.3991
1057.3096
B
Pelargonidin 3-O-rutinoside
579.1604
121.0296
294.0500
295.0584
303.0484
325.0685
337.0680
405.0920
423.1012
447.1006
580.1638
655.1523
656.1556
817.1993
819.1988
1023.2511
1157.3040
1185.3047
C
Pelargonidin 3,5-O-diglucoside
595.1489
271.0562
283.0562
313.0656
337.0643
433.1028
565.1372
596.1520
655.1453
817.1903
1023.2402
1185.2911
D
Cyanidin 3-O-galactoside
595.1566
187.0604
205.0702
207.0637
247.0804
287.0515
325.0665
341.0620
353.0614
409.0861
421.0838
449.1015
463.0925
553.1619
577.1425
596.1579
%
m/z

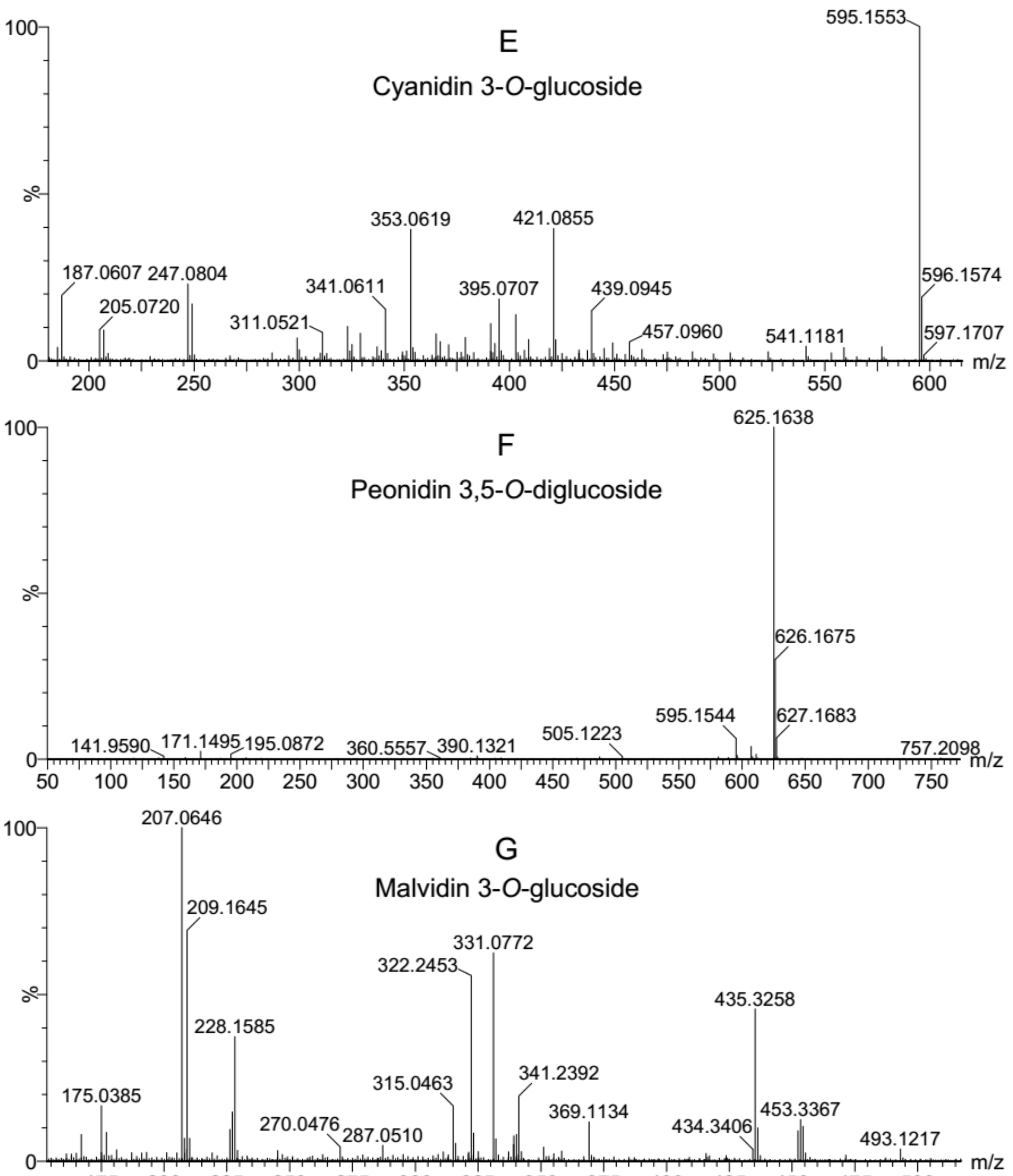

Figure S1 UPLC-ESI/MS/MS of anthocyanins in the peels of pooled *D. officinale* green and red stems. Precursor-ion analysis of (A) delphinidin 3,5-*O*-diglucoside; (B) pelargonidin 3-*O*-rutinoside; (C) pelargonidin 3,5-*O*-diglucoside; (D) cyanidin 3-*O*-galactoside; (E) cyanidin 3-*O*-glucoside; (F) peonidin 3,5-*O*-diglucoside; (G) malvidin 3-*O*-glucoside.

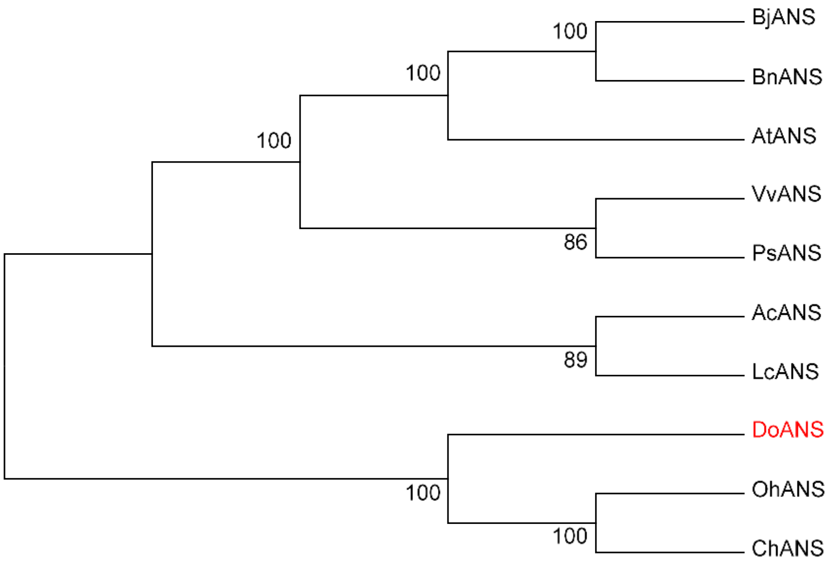

Figure S2 Phylogenetic analysis of *D. officinale* DoANS with reported ANSs from other plants. Neighbor-joining phylogenetic analysis was conducted using MEGA5.0 with 1000 bootstrap replicates. ANS proteins used for phylogenetic profiling alignment are: AcANS (*Allium cepa*, EF192475), AtANS (*Arabidopsis thaliana*, JF681791), BjANS (*Brassica juncea*, EU927147), BnANS (*Brassica napus*, KF250411), ChANS (*Cymbidium dayanum*, KM186177), LcANS (*Lycoris chinensis*, KC131464), OhANS (*Oncidium hybridum*, EF570114), PsANS (*Prunus salicina*, JN560957) and VvANS (*Vitis vinifera*, EU156063).

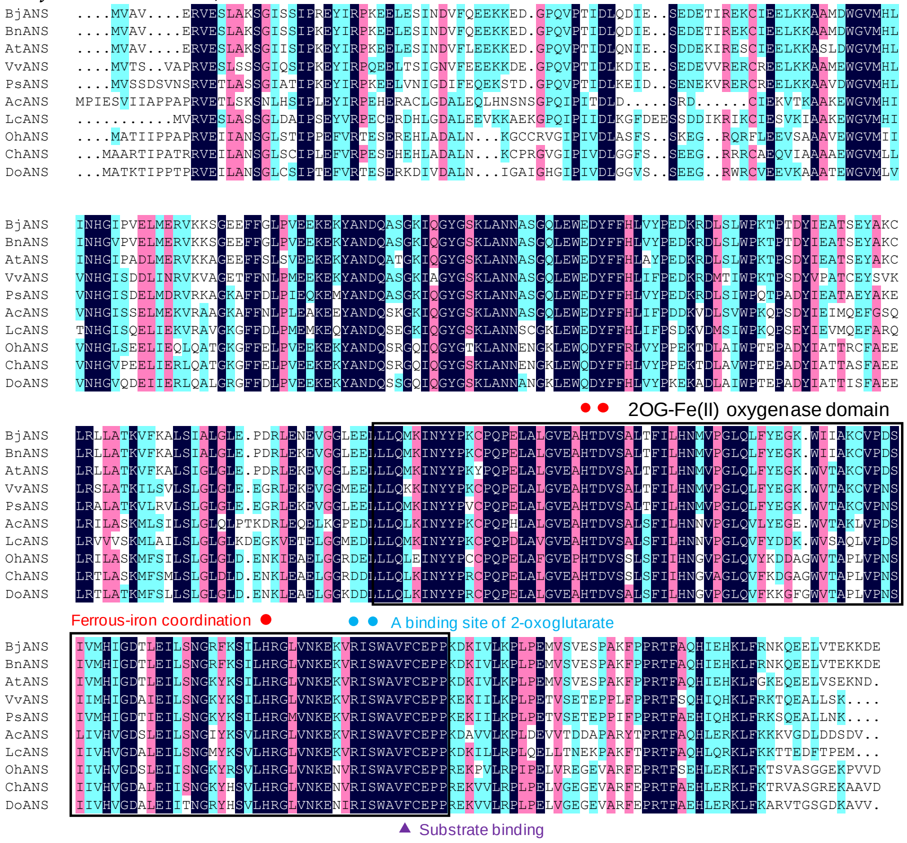

Figure S3 Sequence alignment of *D. officinale* DoANS with known ANSs from other plants using ClustalX

multiple alignment tool. Residues framed in a rectangle are postulated to be the 2OG-Fe(II) oxygenase domain. Red circles indicate residues for ferrous-iron coordination. Blue circles indicate residues for a binding site of 2-oxoglutarate. Purple arrow-head indicates residue for substrate binding. ANS proteins used for phylogenetic profiling alignment are: AcANS (*Allium cepa*, EF192475), AtANS (*Arabidopsis thaliana*, JF681791), BjANS (*Brassica juncea*, EU927147), BnANS (*Brassica napus*, KF250411), ChANS (*Cymbidium dayanum*, KM186177), LcANS (*Lycoris chinensis*, KC131464), OhANS (*Oncidium hybridum*, EF570114), PsANS (*Prunus salicina*, JN560957) and VvANS (*Vitis vinifera*, EU156063).

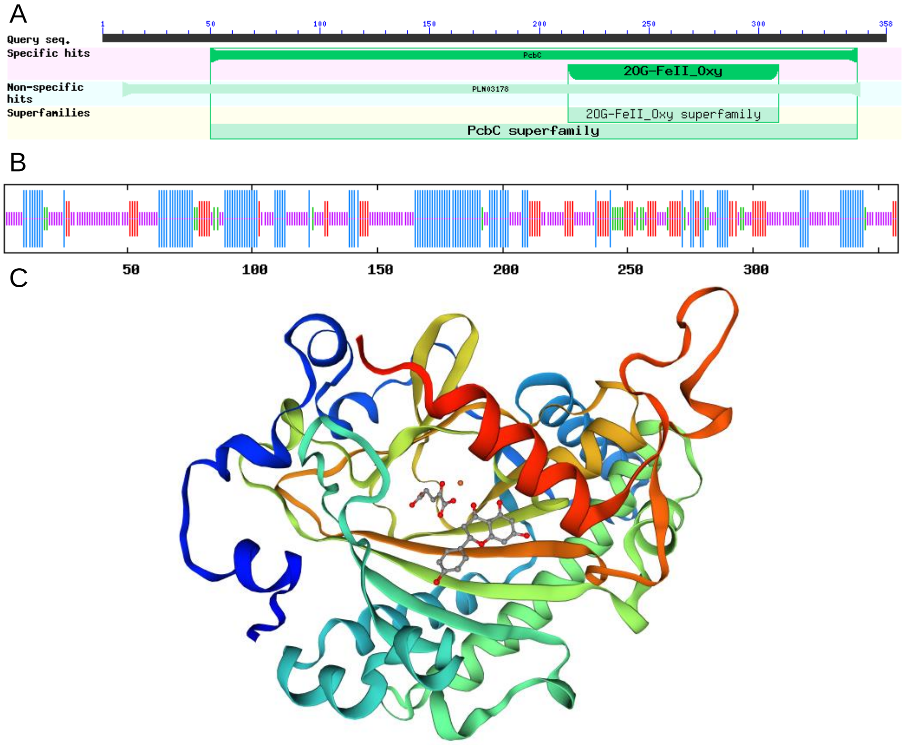

Figure S4 The structural characteristics of *D. officinale* anthocyanin synthase (DoANS). (A) Conserved functional domain annotation; (B) Secondary protein structure of DoANS: α-helix, extended strand and random coil are represented by the longest, second longest and shortest vertical bars, respectively; (C) Tertiary structure of the characteristic functional region, showing the OG-Fe(II) oxygenase domain in the form of a carbon frame.

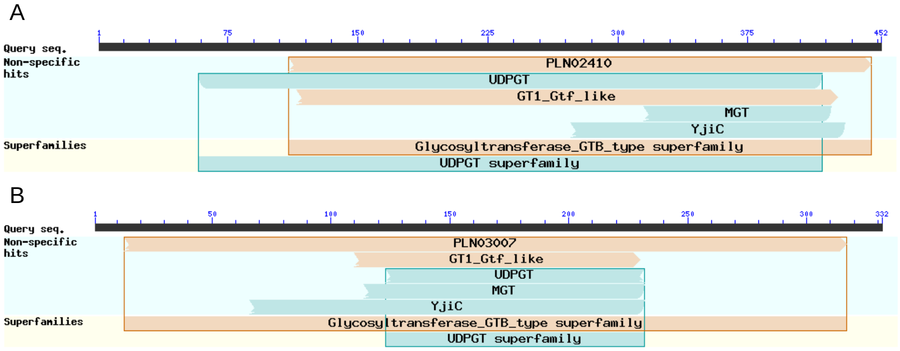

Figure S5 Conserved functional domain of UDP-glucose flavonoid 3-O-glucosyl transferase proteins in *D. officinale*. (A) Conserved domain annotation of DoUFGT1; (B) Conserved domain annotation of DoUFGT2.

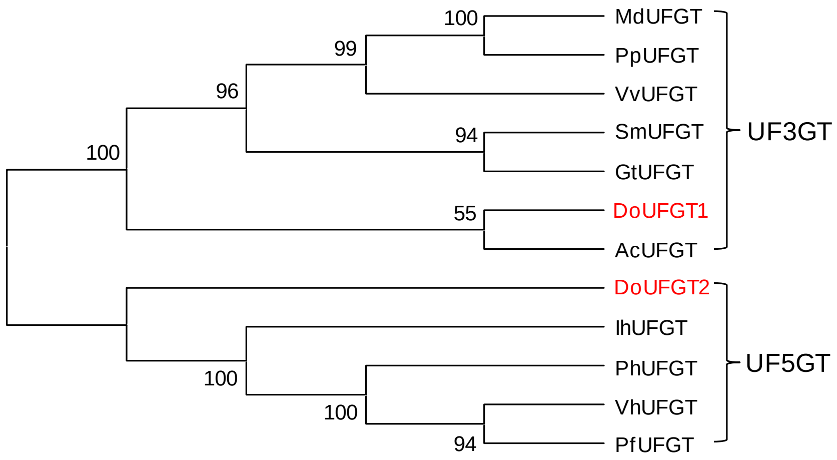

Figure S6 Sequence alignment of *D. officinale* DoUFGT1 and DoUFGT2 with known UFGTs from other plants using ClustalX multiple sequence alignment tool. UF3GT indicates glucosylation of the anthocyanidins at the 3-*O*-position, while UF5GT indicates glucosylation of the anthocyanidins at the 5-*O*-position. UFGT proteins used for phylogenetic profiling alignment are: AcUFGT (*Allium cepa*, KY273099); GtUFGT (*Gentiana triflora*, Q96493); IhUFGT (*Iris hollandica*, AB113664); MdUFGT (*Malus domestica*, AF117267); PfUFGT (*Perilla frutescens*, AB013596); PhUFGT (*Petunia hybrida*, AB027455); PpUFGT (*Prunus persica*, JX149550); SmUFGT (*Solanum melongena*, Q43641); VhUFGT (*Verbena hybrida*, AB013598); VvUFGT (*Vitis vinifera*, DQ513314).

References

1. Wrolstad, R.E.; Putnam, T.P.; Varseveld, G.W. Color quality of frozen strawberries: effect of anthocyanin, pH, total acidity and ascorbic acid variability. *J Food Sci* **1970**, *35*, 448-452.
2. Lichtenthaler, H.K.; Wellburn, A.R. Determinations of total carotenoids and chlorophylls A and B of leaf extracts in different solvents. *Biochem Soc Trans* **1983**, *11*, 591-592.
3. Fu, X.M.; Cheng, S.H.; Liao, Y.Y.; Huang, B.Z.; Du, B.; Zeng, W.; Jiang, Y.M.; Duan, X.W.; Yang, Z.Y. Comparative analysis of pigments in red and yellow banana fruit. *Food Chem* **2018**, *239*, 1009-1018.
